# Concurrent Stage III Unresectable Duodenal Adenocarcinoma and Metastatic Gastrointestinal Stromal Tumor Treated With Combination of Imatinib and mFOLFIRI

**DOI:** 10.7759/cureus.58248

**Published:** 2024-04-14

**Authors:** Amith Rao, Rohit Rao, Megan K Taylor, Mohammad Khreiss, Junaid Arshad

**Affiliations:** 1 Department of Internal Medicine, Banner Health, Tucson, USA; 2 Department of Internal Medicine, University Hospitals Cleveland Medical Center, Cleveland, USA; 3 Department of Internal Medicine, University of Arizona College of Medicine, Tucson, USA; 4 Department of Surgical Oncology, University of Arizona College of Medicine, Tucson, USA; 5 Department of Medical Oncology, University of Arizona Cancer Center, Tucson, USA

**Keywords:** imatinib, gastrointestinal stromal tumor (gist), mfolfiri, tyrosine kinase inhibitors (tki), duodenal adenocarcinoma

## Abstract

Cases of concurrent duodenal adenocarcinoma and gastrointestinal stromal tumors (GISTs) are rare, and only a few have been reported. While some cases of other synchronous primary tumors with GIST have been reported, no shared mutations have been consistently found, creating challenges in selecting chemotherapy in cases of inoperable tumors. Here, we presented a case of a stage IIIA locally advanced/unresectable duodenal adenocarcinoma with concurrent metastatic small bowel GIST successfully being treated with combined imatinib and modified folinic acid, 5-fluorouracil, and irinotecan (mFOLFIRI) regimen.

## Introduction

Gastrointestinal stromal tumors (GISTs) make up approximately 1% of all gastrointestinal cancers, with a five-year overall survival rate of 85% [1]. However, this survival rate drops to 52% when considering cases where metastatic disease has been noted on initial diagnosis [1]. In the literature, about 14-33% of GISTs co-occur with other cancers [2]. Synchronous primary tumors with GIST have been reported with peritoneal mesothelioma [2], colorectal cancer [3,4], ovarian and lung cancer [4], renal cell carcinoma [5], gastric adenocarcinoma [6-8], pancreatic cancer [9], intrahepatic cholangiocarcinoma [10], melanoma [11], neuroendocrine tumor [11], ampullary carcinoma [11], liposarcoma [12], distinct GISTs [13], and gallbladder carcinoma [14]. Previous literature has focused on surgical resection of either the primary tumor or GIST with subsequent treatment of the other tumor. However, limited data exists outlining proper procedure in cases of an inoperable primary GI malignancy and a concurrent inoperable metastatic GIST. First-line medical therapy for small bowel adenocarcinoma is typically oxaliplatin-based regimens such as capecitabine plus oxaliplatin (CAPOX) or leucovorin, 5-fluorouracil, oxaliplatin (FOLFOX) [15]; however, most patients with GIST are treated with imatinib or other tyrosine kinase inhibitors (TKIs) [15,16]. Concurrent treatment has largely been avoided in previous studies due to risks of severe neutropenia on a combined regimen of a TKI and standard chemotherapy regimens [15-17]. Here, we will outline the successful ongoing management of stage IIIA locally advanced/unresectable duodenal adenocarcinoma with concurrent metastatic small intestinal GIST with a combined TKI and chemotherapy regimen on a patient who initially failed standard of care FOLFOX therapy.

## Case presentation

**Figure 1:**
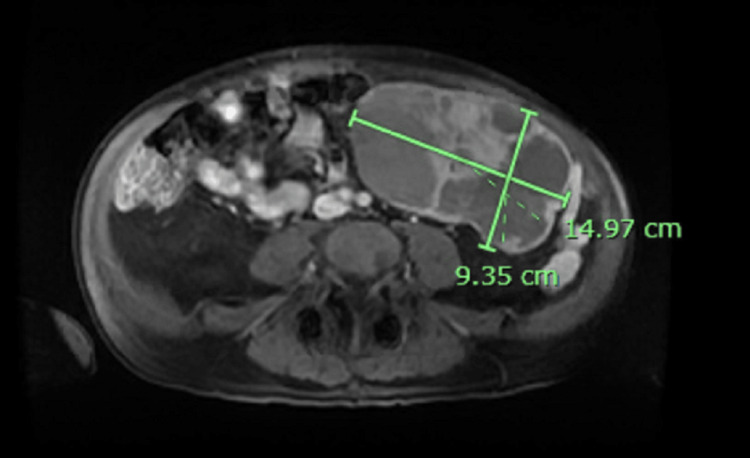
MRCP showing 14.97×9.35 cm biopsy confirmed gastrointestinal stromal tumor. MRCP: magnetic resonance cholangiopancreatography

The patient is a 70-year-old male with hypertension and tobacco use (20-pack-year history) who initially presented with concerns of abdominal pain, jaundice, nausea, and vomiting for three weeks. Symptoms had been slowly worsening over these three weeks but acutely worsened in the 24 hours prior to the ED presentation. The patient had no other medical history or family history of malignancy. The patient had CT abdomen and pelvis without contrast done showing moderate intra and extrahepatic biliary duct dilatation without filling defect and enhancement at the ampulla. To better characterize mass, magnetic resonance cholangiopancreatography (MRCP) was then done, showing mass-like thickening of the third portion of the duodenum with biliary duct dilatation along with a separate mixed cystic and solid mass of the left mid abdomen measuring 14.0×9.7×12 cm and scattered mesenteric lymph nodes and peritoneal implants (Figure 1).

**Figure 2:**
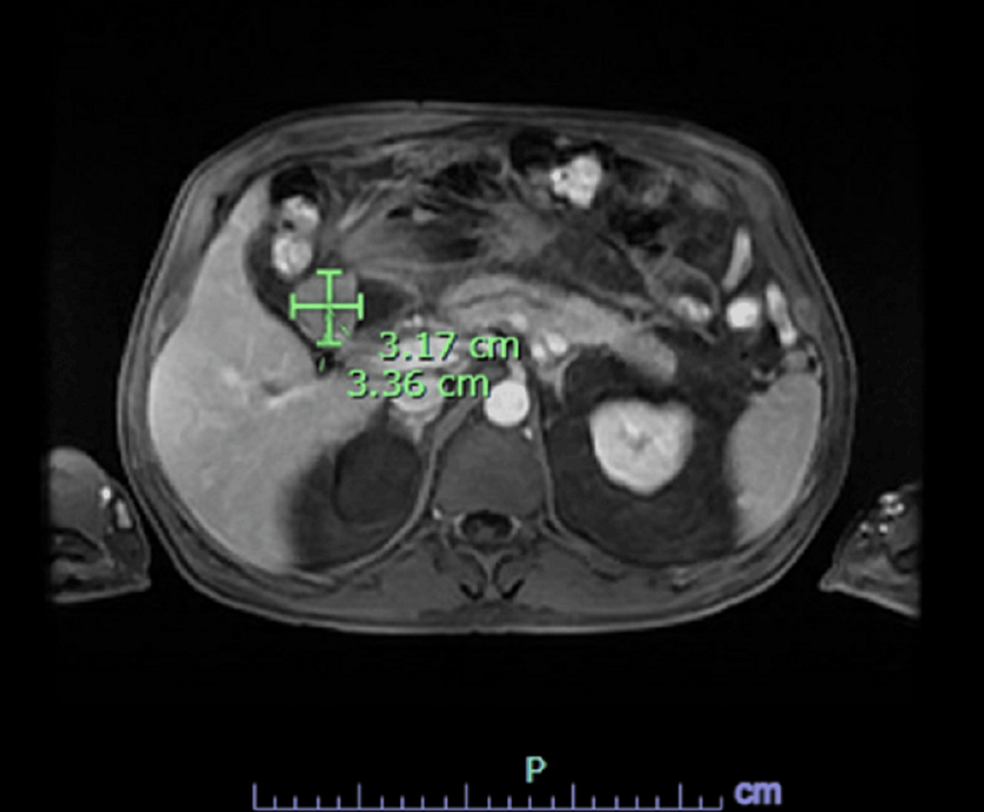
MR abdomen and pelvis with contrast showing 3.36×3.17 cm semiannular duodenal mass s/p stent placement.

The patient had ERCP with biopsy confirming moderately differentiated duodenal adenocarcinoma. Next-generation sequencing (NGS) testing showed pathogenic KIT exon 11 (pD579 del) mutation. Subsequent endoscopy showed severe acquired duodenal stenosis, and a metal 22 mm × 9 cm WallFlex stent (Marlborough, MA: Boston Scientific) was placed (Figure 2).

**Figure 3:**
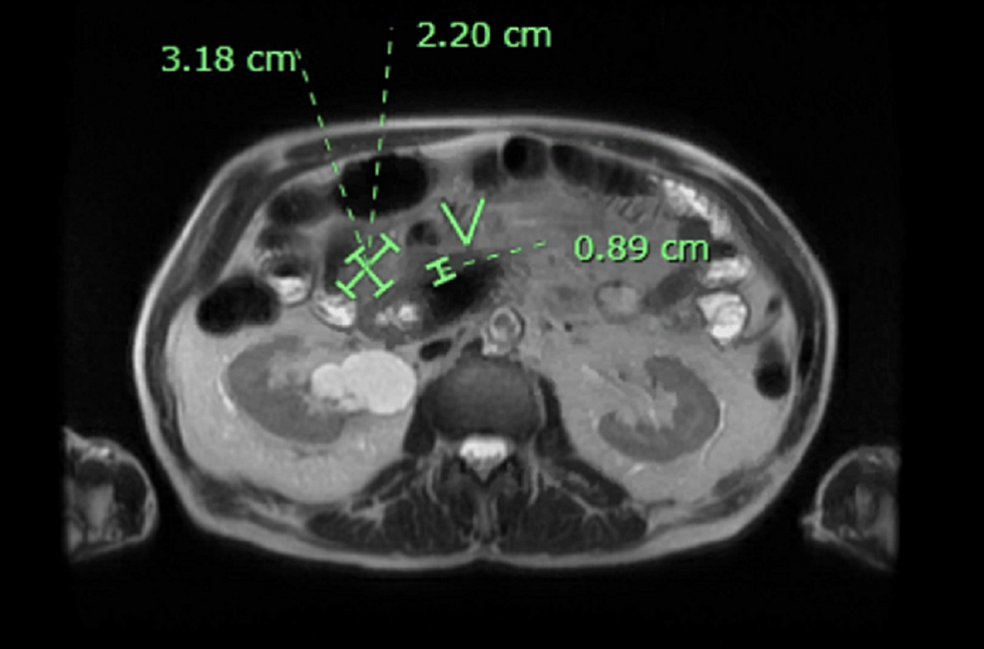
MR abdomen and pelvis with contrast showing 3.18×2.20 cm peritoneal implants, biopsy confirmed metastatic GIST. GIST: gastrointestinal stromal tumor

The patient was staged with CT thorax without contrast, showing evidence of scattered 4 mm pulmonary nodules without evidence of metastatic disease. The case was discussed in the tumor board, and the decision was made to pursue exploratory laparotomy with biopsy of peritoneal implants (mesenteric lymph nodes were too small to be reliably biopsied) to ascertain pathological nature of metastatic disease. The biopsy results confirmed metastatic gastrointestinal stromal tumor (GIST) (Figure 3).

Molecular typing through NGS testing of GIST showed KRAS pathogenic variant exon 2, p.G12A, HER2 negative (1+), and a pathogenic KIT exon 11 (pD579 del) mutation. PET CT confirmed the presence of locally advanced duodenal adenocarcinoma with no other areas of metastasis. Surgical oncology was consulted and determined duodenal adenocarcinoma was unresectable based on the location of the tumor abutting into the superior mesenteric artery.

The decision was then made to pursue medical management, and the patient was initially started on FOLFOX chemotherapy regimen for the management of duodenal adenocarcinoma with intent of curative resection in the setting of chemotherapy response. The treatment for the small intestinal GIST (imatinib) was held due to concern for combined therapy causing severe neutropenia, asymptomatic slow-growing nature of GIST, age of the patient, and desire to treat the more severe malignancy.

**Figure 4:**
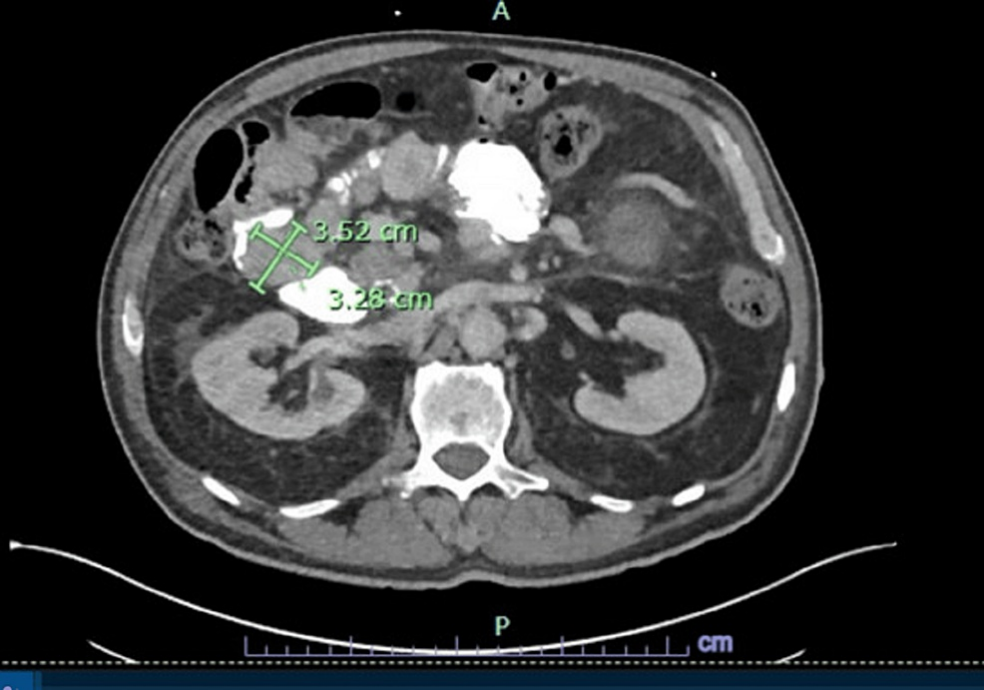
Worsening peritoneal implants on repeat CT abdomen/pelvis with contrast post-small bowel GIST resection in hospital (3.2×3.52 cm).

The patient completed four cycles of FOLFOX with no interval improvement on follow-up scans of duodenal adenocarcinoma and was switched to mFOLFIRI (leucovorin, 5-fluorouracil, irinotecan) regimen to avoid end-organ damage and neuropathic side effects of oxaliplatin in the setting of uncertain response to chemotherapy. This subsequent treatment course was complicated by incidence of small bowel volvulus and methicillin-sensitive Staphylococcus aureus (MSSA) bacteremia, requiring an urgent resection of the small bowel GIST. A repeat CT abdomen and pelvis was done in the hospital and the patient was found to have worsening peritoneal implants (Figure 4).

The decision was then made to start low-dose imatinib 200 mg daily with mFOLFIRI treatment to treat both cancers due to worsening implants. The patient has completed 34 cycles of mFOLFIRI with low-dose imatinib maintenance therapy for 18 months. The dose of irinotecan was reduced to 150 mg from 180 mg, and the initial 5-fluorouracil (5-FU) bolus was removed. This course was complicated by acute cholangitis requiring replacement of biliary stent and treatment seven months after treatment; however, peritoneal implants have remained stable with no progression of either malignancy and no severe neutropenia (ANC<500), bleeding, or end-organ damage. The ongoing treatment plan is to continue maintenance therapy with imatinib and mFOLFIRI to control the spread/growth of both malignancies with repeat MR abdomen and pelvis every three months and ongoing outpatient follow-up every two to three weeks with repeat CBC, comprehensive metabolic panel {CMP}, and carcinoembryonic antigen {CEA}.

## Discussion

We describe a patient with synchronous metastatic GIST and duodenal adenocarcinoma treated with concurrent imatinib and mFOLFIRI. To our knowledge, we are the first to report the use of this regimen to treat both cancers simultaneously.

Medical treatment of GISTs has focused on targeting the oncogenic c-kit receptor which is thought to drive the outgrowth of these tumors [13]. This is done using tyrosine kinase inhibitors (TKIs). A few case reports have discussed the targeting of c-kit in the setting of synchronous GISTs and adenocarcinomas. One case report demonstrated that apatinib monotherapy can induce partial response in synchronous GIST and primary gastric adenocarcinoma following the progression of both tumors on a combination of imatinib and S1 (oral fluoropyrimidine) therapy [7]. In another case, adjuvant CAPOX + 400 mg imatinib showed efficacy in limiting the progression of disease in synchronous rectal cancer and GIST [15]. In the present case, we had to perform treatment of a synchronous duodenal adenocarcinoma and GIST metastatic to the peritoneum in a patient who had already failed FOLFOX and mFOLFIRI therapy. The molecular landscape of both tumors was tested, and interestingly both tumors had a pathogenic KIT exon 11 mutation. This mutation has been shown in prior studies to correlate with sensitivity to imatinib in metastatic GIST [16]. However, the significance of this mutation in duodenal adenocarcinoma is unknown and requires more study.

One challenge of combination of conventional regimens and Kit inhibitors is maintaining efficacy against multiple tumors while avoiding synergistic toxicity. Due to the rarity of small bowel tumors, most of the data on the safety of imatinib + chemotherapy regimens arise from colorectal cancer, in which FOLFOX, FOLFIRI, and XELOX (capecitabine plus oxaliplatin) are used as backbone chemotherapy regimens. The combination of imatinib and mFOLFOX6-bevacizumab chemotherapy in colorectal cancer using three different dose levels of imatinib (400, 600, and 800 mg/day) demonstrated a high incidence of grade III/IV neutropenia, and only modest anti-tumor response at 400 and 600 mg dose levels for imatinib [17]. On the other hand, dose-reduced XELOX + bevacizumab + 300 mg imatinib showed a better response profile with only 2/49 patients with grade III neutropenia and an overall response rate of 45% [18]. For our patient, we decided to use mFOLFIRI as a backbone for combination chemotherapy after the failure of FOLFOX. As the rate of neutropenia has been reported to be higher with mFOLFIRI (21.8%) [19] compared to XELOX (7%, grade III/IV neutropenia) [20], we elected to proceed with a lower dose of 200 mg daily imatinib and a reduced dose of 150 mg irinotecan (standard dose 180 mg). At 18 months out, our patient’s disease remains stable on this combination therapy with no significant severe hematological toxicities and favorable overall survival.

This case shows that a combined regimen of imatinib/mFOLFIRI can be used as an option if both malignancies need to be treated concurrently. Our patient had worsening peritoneal implants after resection of primary GIST in an emergency situation (small bowel volvulus), necessitating treatment of both malignancies. The risks of grade 3/4 neutropenia are still very real; however, the standard of care should prioritize treatment of the more aggressive malignancy first in cases of concurrent primary tumors with GIST, as GISTs are often very slow-growing and asymptomatic.

## Conclusions

In this report, we described the successful treatment of a synchronous metastatic GIST and small bowel adenocarcinoma with a novel mFOLFIRI-low dose imatinib regimen. While synchronous GIST and other malignancies are not unheard of, there still is a need for research on chemotherapy + TKI regimens that adequately address both diseases. We show that mFOLFIRI + low dose imatinib can be a safe and effective regimen in treating synchronous small bowel adenocarcinoma and GIST. The efficacy of this regimen may offer hope for treatment of other synchronous GI malignancies that co-occur with GIST such as colorectal and gastric cancer.
